# Global Burden of Pancreatic Cancer Attributable to High Body-Mass Index in 204 Countries and Territories, 1990–2019

**DOI:** 10.3390/cancers16040719

**Published:** 2024-02-08

**Authors:** Irena Ilic, Milena Ilic

**Affiliations:** 1 Faculty of Medicine, University of Belgrade, 11000 Belgrade, Serbia; 2 Department of Epidemiology, Faculty of Medical Sciences, University of Kragujevac, 34000 Kragujevac, Serbia; drmilenailic@yahoo.com

**Keywords:** pancreatic cancer, burden, body mass index, trends

## Abstract

**Simple Summary:**

The burden of pancreatic cancer attributable to a high body mass index (BMI) increased significantly for both sexes, in all ages, across all socio-demographic index (SDI) quintiles, and all GBD regions. There were apparent international variations in ASRs both for mortality and DALYs for pancreatic cancer that could be attributed to a high BMI in 2019: higher ASRs were observed in females, in the high SDI region than in other SDI regions, and in the Central Europe region than in other GBD regions. Growth trends in pancreatic cancer burden that could be attributed to a high BMI were faster in the low than in the high SDI region.

**Abstract:**

(1) Background: This study aimed to assess the global burden of pancreatic cancer attributable to a high BMI in 1990–2019. (2) Methods: An ecological study was carried out. Data about deaths and Disability-Adjusted Life Years (DALYs) for pancreatic cancer were extracted from the Global Burden of Disease (GBD) study. The age-standardized rates (ASRs, per 100,000) were presented. In order to determine trends of pancreatic cancer burden, joinpoint regression analysis was used to calculate the average annual percent change (AAPC). (3) Results: The highest ASRs of DALYs of pancreatic cancer were found in the United Arab Emirates (47.5 per 100,000), followed by countries with about 25.0 per 100,000 (such as Hungary, Czechia, and Montenegro). From 1990 to 2019, the ASRs of deaths and DALYs of pancreatic cancer attributable to a high BMI significantly increased (*p* < 0.001) for both sexes in all ages, and across all SDI quintiles and all GBD regions. The highest fraction of DALYs attributable to a high BMI was found in the United States of America and China (equally about 15.0%), followed by the Russian Federation, India, Germany, and Brazil (about 5.0%, equally). (4) Conclusions: Further analytical epidemiological studies are necessary to elucidate the relationship between pancreatic cancer and a high BMI.

## 1. Introduction

According to the GLOBOCAN 2020 estimates, pancreatic cancer is the 12th most common cancer in both sexes in the world, with the highest incidence rates reported in Europe, North America, and Australia/Oceania [1,2]. The increase in pancreatic cancer incidence and mortality was seen across almost all countries worldwide from 1991 onwards [3,4]. In contrast, favorable trends of pancreatic cancer mortality were reported only in Canada and Mexico. A comparative mortality study covering 28 countries of the European Union predicted that pancreatic cancer will surpass breast cancer by 2025 (there will be more deaths from pancreatic cancer than from breast cancer, while the number of pancreatic cancer deaths will increase by 25%), whereby pancreatic cancer will be the third leading cause of cancer death by 2025 (after lung cancer and colorectal cancer) [5]. It remains a question of what effect the COVID-19 pandemic will have on the burden of pancreatic cancer, since previous findings were not consistent: while the findings of a retrospective multicenter cohort study showed that there were no changes in clinical presentation, treatment strategies and survival of pancreatic cancer during the SARS-CoV-2 outbreak [6], some studies reported an increase in tumors localized in the tail of the pancreas and that patients presented with much greater advancement of the disease in 2020 compared to 2019 [7], and that a delay of only 3 months for surgery in stage II or III pancreatic cancer was associated with an over 17% reduction in long-term survival [8].

Some authors consider the increase in the frequency of pancreatic cancer to largely be a reflection of the aging and growth of our population in the world [9]. On the one hand, the rise in pancreatic cancer mortality could be considered a consequence of decline in mortality from other common malignancies in previous decades, due to the implementation of screening and improvements in treatment (in breast cancer, stomach and colorectal cancer), as well as improved cancer registration practices. However, epidemiological studies have reported that geographic differences in pancreatic cancer burden might be explained by variations in the prevalence of some underlying risk factors such as smoking, diabetes, alcohol use, dietary factors, and obesity [10,11]. Globally, from 1990 to 2019, the largest increase in risk exposure was related to a high body mass index [12]. Still, to date, the etiology of pancreatic cancer remains insufficiently elucidated.

Considering the numerous and enormous challenges in achieving the goals that the World Health Organization (WHO) and the United Nations Sustainable Development Goals (UNSDGs) declared in 2015, that is, the reduction of the premature mortality rate attributed to cancer by one third by 2030 [13], a better understanding of the burden patterns of pancreatic cancer worldwide can provide further insights into the etiology of this disease and the possible ways for a more effective prevention and control of it. The main purpose of this manuscript was to assess international patterns in pancreatic cancer burden attributable to a high body mass index in the recent three decades.

## 2. Materials and Methods

### 2.1. Study Design

This ecological study comprised the annual underlying cause of death data to describe the burden of pancreatic cancer worldwide, i.e., across 204 countries and territories.

### 2.2. Data Source

Pancreatic cancer cases data were extracted from databases of the Global Burden of Disease (GBD) 2019 study [14]. The GBD study is the largest global scientific effort conducted to estimate and compare the magnitudes as well as trends of diseases and risk factors across countries.

The GBD databases provide high-quality estimates of the burden of pancreatic cancer worldwide, and also provide an assessment of the contribution of certain risk factors to the burden of pancreatic cancer [14]. For the assessment of pancreatic cancer mortality, disability and associated risk factors, the GBD 2019 study obtained data from multiple data sources, including national vital statistics, cancer registries, verbal autopsy reports, national health surveys, censuses, and published studies. Pancreatic cancer presented in this study includes malignant neoplasms defined by the International Statistical Classification of Diseases and Related Health Problems, Ninth Revision (ICD-9) as codes 157–157.9, or by the Tenth Revision (ICD-10) as codes C25–C25.9, containing primary malignancy of the pancreas [14,15]. The GBD 2019 study is consistent with the best reporting practices for studies that calculate health estimates, according to the Guidelines for Accurate and Transparent Health Estimates Reporting (GATHER) [16]. The GBD 2019 study reported data for pancreatic cancer for global and regional level (including 21 GBD regions and 5 socio-demographic index (SDI) quintiles), i.e., 204 countries and territories. The GBD estimates for pancreatic cancer are available for every age group, sex, location, and year. According to the GBD comparative risk assessment framework for computing the fraction of the burden of disease attributable to some risk factor, a high body mass index (BMI) is indicated as a risk factor for pancreatic cancer [17].

### 2.3. Study Variables and Measures

This manuscript presents pancreatic cancer burden estimates, including deaths and Disability-Adjusted Life Years (DALYs). DALYs for pancreatic cancer are calculated as the sum of the years of life lost due to premature mortality from that cause and the years of healthy life lost due to disability resulting from this cause. One DALY represents the loss of the equivalent of one year of full health. For pancreatic cancer burden, all figures were adjusted by age and presented as age-standardized rates (ASRs, per 100,000 persons) calculated by the direct method of standardization, using the GBD 2019 standard population [14]. The results for countries/territories that had less than 100,000 inhabitants (such as Andorra, American Samoa, Antigua and Barbuda, Cook Islands, Dominica, Greenland, Marshall Islands, Monaco, Nauru, Niue, Northern Mariana Islands, Palau, Saint Kitts and Nevis, San Marino, Tokelau, and Tuvalu) are shown, but they were not considered in comparisons due to the consequent instability of the rates.

The socio-demographic index, a summary measure of the level of development of a country, was calculated based on three indicators: income per capita, average years of schooling in ages 15 and older, and total fertility rate of females under age 25 [14]. The SDI is expressed on a scale of 0.0 (the worst, i.e., lowest SDI) to 1.0 (the best, i.e., highest SDI), with quintiles used to categorize low, low-middle, middle, high-middle, and high SDI countries. The GBD risk factor hierarchy and accompanying exposure defined the exposure of a high BMI (>25 kg/m^2^) using theoretical minimum risk exposure level at a BMI value of 20–25 kg/m^2^ [13].

### 2.4. Statistical Analysis

Temporal trends in the burden of pancreatic cancer were evaluated using the Joinpoint Regression Analysis Software (Version 4.9.0.0; National Cancer Institute, Bethesda, MD, USA—March 2021) proposed by Kim et al. [18]. In order to assess the magnitude and direction of trends in burden of pancreatic cancer, the joinpoint regression analysis detected a “joinpoint”, i.e., a temporal point at which there was a significant change in the frequency of pancreatic cancer. The permutation test was used for multiple comparisons (at 4499 permutations) [18]. For this analysis, a minimum of zero joinpoints (one line segment) and a maximum of five joinpoints (six line segments) were allowed for each model. This study presented only the results based on the minimum number of joinpoints (i.e., trend as a one straight line). The average annual percent change (AAPC) over the whole observed period was computed; for each AAPC estimate, the corresponding 95% confidence interval (95%CI) was calculated [19]. For describing the direction of temporal trends, the term “significant change” (increase or decrease) was used in order to signify that the slope of the trend was statistically significant (*p* < 0.05). Analysis was performed by sex and age (<20, 20–24, …, 90–94, 95+). Also, in this study, the proportion (%) of ASRs of deaths and DALYs for pancreatic cancer attributable to a high BMI in 204 countries and territories in 2019 was presented. Additionally, the correlation of the ASRs for DALYs of pancreatic cancer attributable to a high BMI with SDI level was determined using linear correlation models and presented with regression curves. The correlation was estimated with Pearson’s correlation coefficient. Statistical significance was accepted at the level of *p* < 0.05; analyses were conducted using SPSS Software (Version 20.0, Chicago, IL, USA).

### 2.5. Ethical Considerations

This study was approved by the Ethics Committee of the Faculty of Medical Sciences, University of Kragujevac (Ref. No.: 01-14321, 13 November 2017), entitled “Epidemiology of the most common health disorders”. The data are fully aggregated, without any identification data, and no patient approvals were required for this study.

## 3. Results

**Figure 1:**
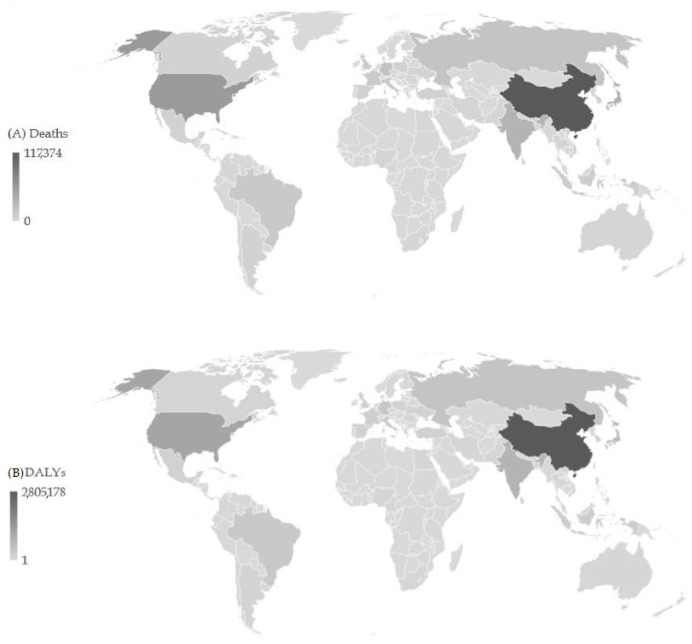
Number of deaths (**A**) and DALYs (**B**) of pancreatic cancer across 204 countries/territories in 2019.

In the 204 countries and territories, the total number of pancreatic cancer deaths in all ages and both sexes was 530,864 in 2019 (Figure 1). Most of the deaths (117,374; 22.1% of the total) were recorded in China, followed by the United States of America (57,448; 10.8% of the total), Japan (37,462; 7.1% of the total), and India (33,546; 6.3% of the total). The least number of pancreatic cancer deaths (<10) in 2019 was recorded in some island countries in Oceania and Africa. The total number of DALYs of pancreatic cancer in all ages and both sexes was 11.5 million in 2019. The highest number of DALYs was found in China (2.8 million; 24.3% of the total), followed by the United States of America (1.1 million; 9.9% of the total). The least number of pancreatic cancer DALYs (<100) in 2019 was recorded in some island countries in Oceania and Africa.

**Figure 2:**
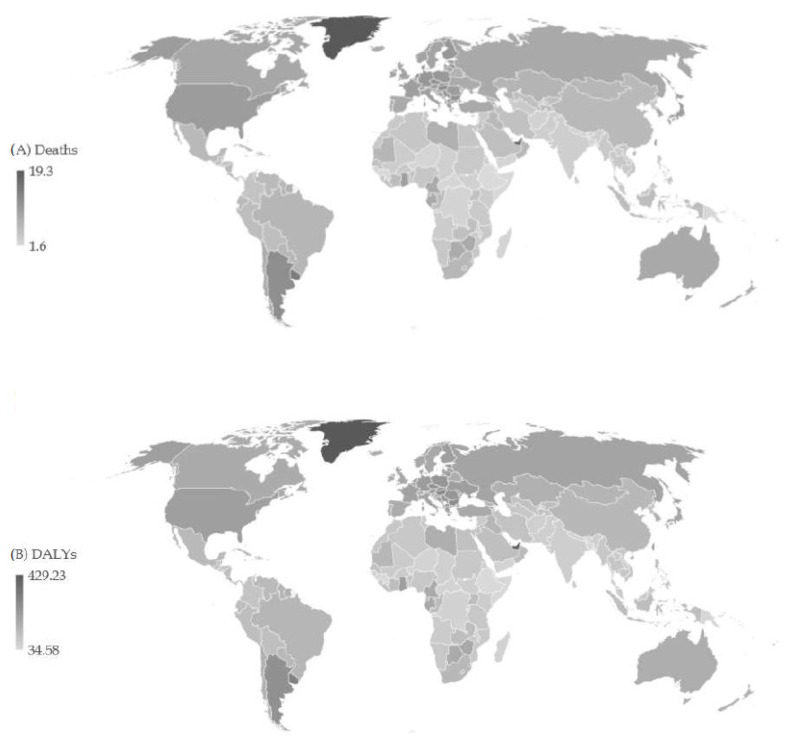
Age-standardized rates (ASRs) of death (**A**) and DALYs (**B**) of pancreatic cancer across 204 countries/territories in 2019.

Globally, the ASR of pancreatic cancer mortality was 6.5 per 100,000 in all ages and both sexes in 2019 (Figure 2). The highest ASRs of mortality were found in the United Arab Emirates (17.6 per 100,000) and Uruguay (14.5 per 100,000), while the lowest rates were reported in Ethiopia and Somalia (equally about 1.6 per 100,000). Also, the highest ASRs of pancreatic cancer DALYs were found in the United Arab Emirates (403.7 per 100,000) and Uruguay (307.1 per 100,000), while the lowest rates were reported in Ethiopia (34.6 per 100,000).

**Figure 3:**
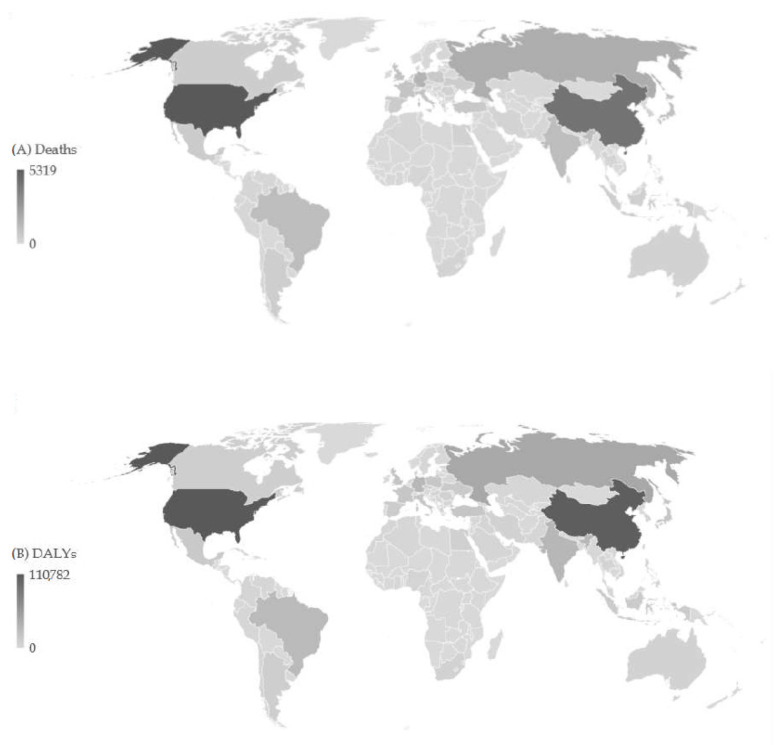
Number of deaths (**A**) and DALYs (**B**) of pancreatic cancer attributable to high body mass index across 204 countries/territories in 2019.

In the 204 countries and territories studied, the total number of pancreatic cancer deaths attributable to a high BMI in all ages and both sexes was 31.905 in 2019 (Figure 3). Most of the deaths (5319; 16.7% of the total) were recorded in the United States of America, followed by China (4236; 13.3% of the total), the Russian Federation (1834; 5.7% of the total), and Germany (1575; 4.9% of the total). The total number of DALYs of pancreatic cancer in all ages and both sexes attributable to a high BMI was 0.7 million in 2019. The highest fraction of DALYs was found in the United States of America and China (equally about 15.0%), followed by the Russian Federation, India, Germany, and Brazil (with a fraction of about 5.0%, equally).

**Figure 4:**
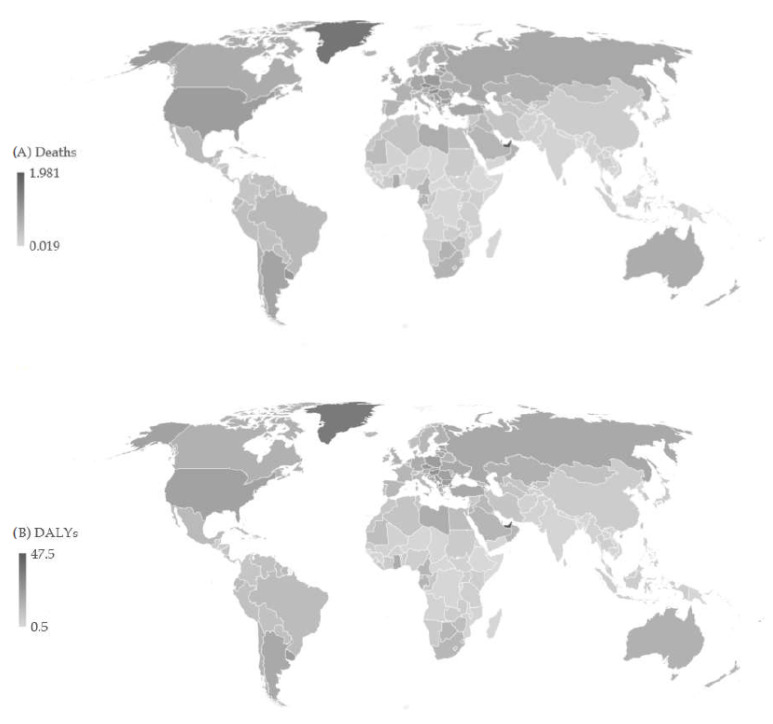
Age-standardized rates (ASRs) of death (**A**) and DALYs (**B**) of pancreatic cancer attributable to a high body mass index across 204 countries/territories in 2019.

Globally, the ASR of pancreatic cancer mortality attributable to a high BMI was 0.4 per 100,000 in all ages and both sexes in 2019 (Figure 4). The highest mortality ASRs were found in the United Arab Emirates (2.0 per 100,000), and then (with about 1.0 per 100,000) in most of the former socialist countries in Central and Eastern Europe (such as Czechia, Hungary, Montenegro, Poland, Estonia, Serbia, Slovakia, Latvia, Bulgaria, and North Macedonia), and Uruguay, Qatar, and the United States of America. The lowest rates were reported in Somalia (0.02 per 100,000). Also, the highest ASRs of pancreatic cancer DALYs were found in the United Arab Emirates (47.5 per 100,000), followed by some countries with about 25.0 per 100,000 (such as Hungary, Czechia, and Montenegro). The lowest rates were reported in Somalia (0.5 per 100,000).

**Figure 5:**
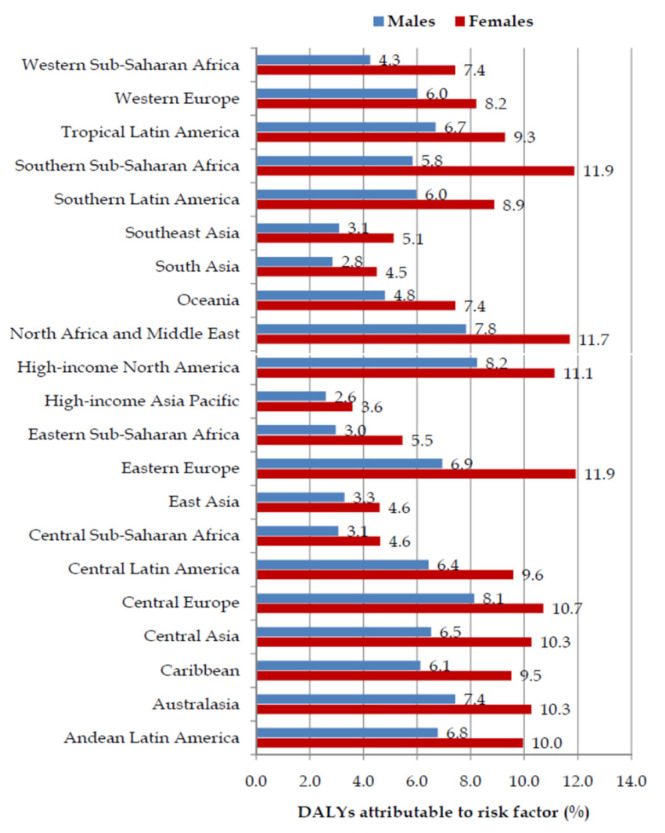
Fraction (%) of pancreatic cancer (DALYs) attributable to a high body mass index by GBD regions and by sex, in 2019.

Fractions (%) of pancreatic cancer DALYs attributable to a high BMI were higher in females than in males in all GBD regions in 2019 (Figure 5). Large disparities (twofold) between males and females in the fraction of pancreatic cancer attributable to a high BMI were observed in the regions of Eastern Europe and Southern Sub-Saharan Africa, while differences by sex were less striking in the high-income Asia-Pacific region.

**Figure 6:**
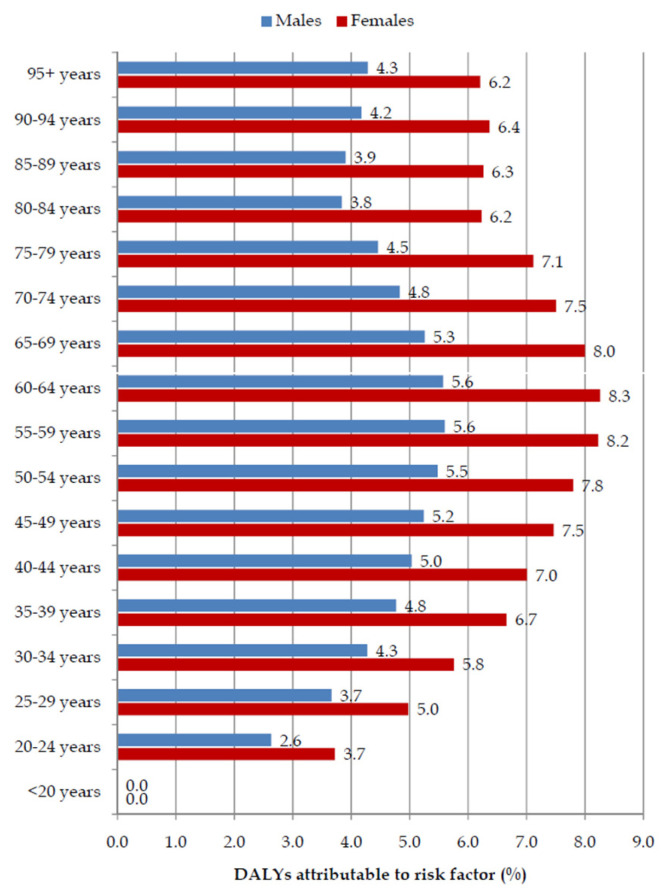
Fraction (%) of pancreatic cancer (DALYs) attributable to high body mass index by age and by sex, in 2019.

Global fractions (%) of pancreatic cancer DALYs attributable to a high BMI were higher in females than in males in all age groups in 2019, with the male to female ratio being 1:1.3 (Figure 6).

**Figure 7:**
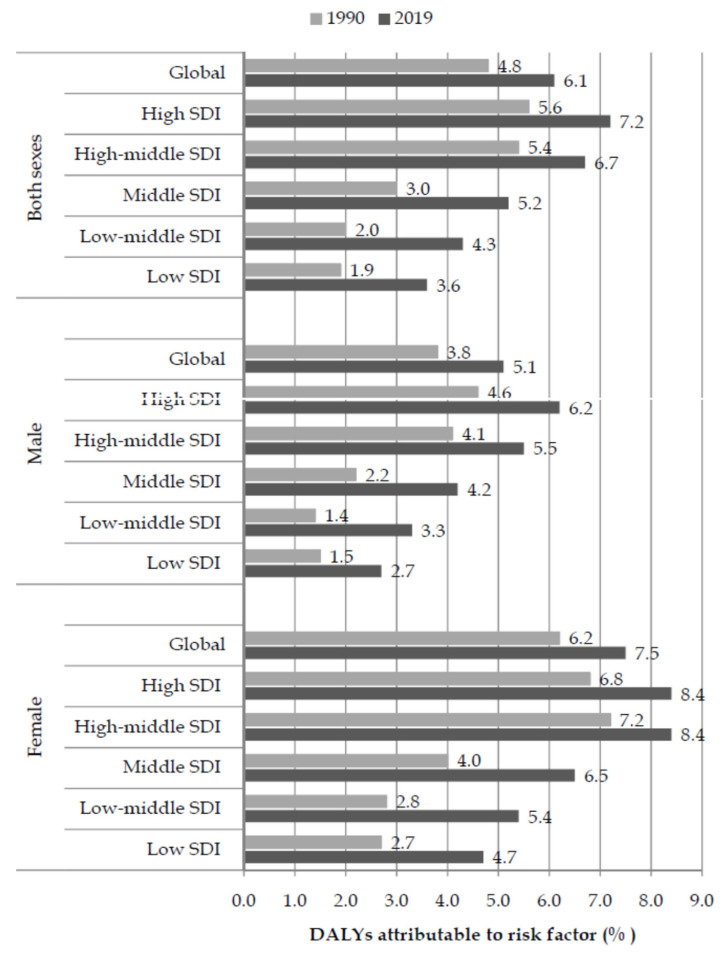
Fraction (%) of pancreatic cancer (DALYs) attributable to a high body mass index by sex and SDI, in 1990 and 2019.

Globally, the fraction of pancreatic cancer DALYs attributable to a high BMI in both sexes together was 4.8% in 1990 and 6.1% in 2019 (Figure 7). Although the fraction of pancreatic cancer attributable to a high BMI was lower in 1990 than in 2019 in both men (3.8% vs. 5.1%) and women (6.2% vs. 7.5%), the values in men in 2019 did not reach the values of woman in 1990. While there were no striking differences for both sexes between 1990 and 2019 in the high-income SDI region, in twofold higher values in the low-income regions twice were described in 2019.

From 1990 to 2019, the ASRs of deaths and DALYs of pancreatic cancer attributable to a high BMI significantly increased (*p* < 0.001) globally for both sexes in all ages, and across all SDI quintiles and all GBD regions (Table 1 and Table 2). There were apparent international variations in ASRs both for mortality and DALYs for pancreatic cancer attributable to a high BMI in 2019: higher ASRs were observed in females than in males, in the high SDI region than in other SDI regions, and in the region of Central Europe than in other GBD regions. The ASRs in the high SDI region were about seven times higher in comparison to the low SDI region. The ASRs in the region of Central Europe were about 10 times higher in comparison to the GBD regions such as Central Sub-Saharan Africa, Eastern Sub-Saharan Africa, Oceania, South Asia, and Southeast Asia. Growth trends in ASRs both for mortality and DALYs for pancreatic cancer attributable to a high BMI from 1990 to 2019 were 3–4 times faster in low SDI and middle SDI countries than in high SDI countries. Growth trends in ASRs both for mortality and DALYs for pancreatic cancer attributable to a high BMI from 1990 to 2019 were 10 times faster in the GBD region of South Asia (equally by AAPC = 6%) than in the high-income Asia-Pacific region (AAPC = 0.6% and AAPC = 0.4%, respectively).

**Figure 8:**
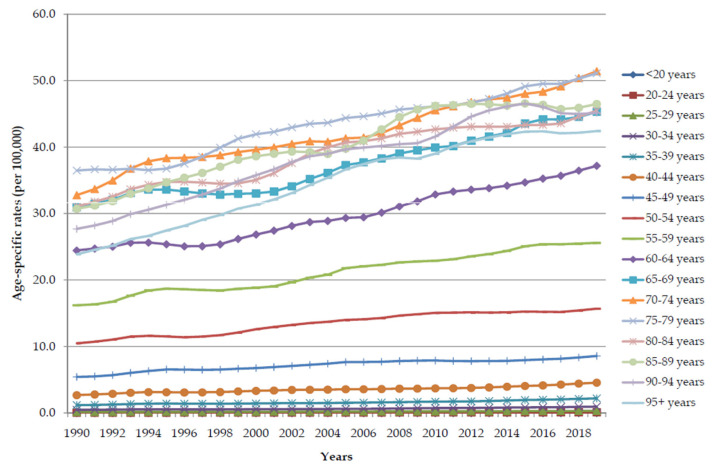
The global trends in age-specific rates of pancreatic cancer DALYs attributable to a high body mass index from 1990 to 2019.

Except for the youngest population (<20 years), the global trends in pancreatic cancer age-specific rates of DALYs attributable to a high BMI in all age groups increased significantly (*p* < 0.001) in both sexes together from 1990 to 2019 (Figure 8). Age-specific rates of pancreatic cancer attributable to a high BMI increased with age and were higher in every age group in 2019 than in 1990. In 1990, the highest age-specific rates were reached at the age of 65–69 years (>30.0 per 100,000), while in 2019, the values of age rates reached higher values (over 30.0 per 100,000) already at the age of 60, with the highest rate (about 51.0 per 100,000) at the age of 70–74.

**Figure 9:**
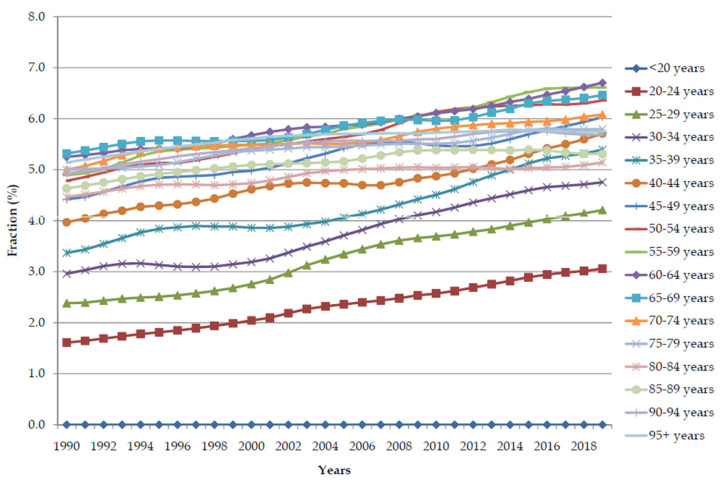
The global trends in fraction (%) of pancreatic cancer DALYs attributable to a high body mass index by age from 1990 to 2019.

Except for the youngest population (<20 years), the global trends in fractions (%) of pancreatic cancer DALYs attributable to a high BMI in all age groups significantly increased (*p* < 0.001) in both sexes together from 1990 to 2019 (Figure 9). The fractions of pancreatic cancer attributable to a high BMI increased with ageing, and were higher in each age group in 2019 than in 1990. In 1990, the highest values of the fraction were reached in the age group of 60–69 years (about 5.3%), while in 2019, the values of the fraction reached higher values (over 5.3%) already in the age group of 35–39 years.

**Figure 10:**
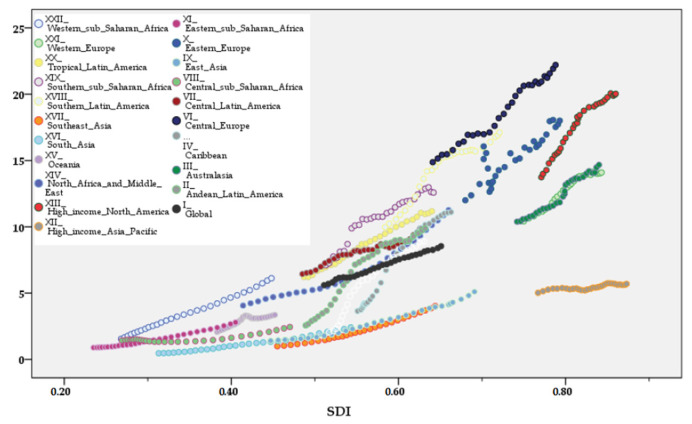
The trend in ASRs of pancreatic cancer DALYs attributable to a high body mass index across 21 GBD regions by SDI from 1990 to 2019.

Pearson’s coefficient showed a significant positive correlation between the ASRs of pancreatic cancer DALYs attributable to a high BMI in all ages and both sexes together as well as SDI from 1990 to 2019 in all GBD regions (*p* < 0.001) (Figure 10).

## 4. Discussion

The burden of pancreatic cancer that could be attributed to a high BMI significantly increased for both sexes and in all ages globally, and across all SDI quintiles and all GBD regions. There were apparent international variations in ASRs both for mortality and DALYs for pancreatic cancer that could be attributed to a high BMI in 2019: higher ASRs were observed in females than in males, in the high SDI region than in other SDI regions, and in the region of Central Europe than in other GBD regions. Growth trends in pancreatic cancer that could be attributed to a high BMI were faster in the low SDI region than in the high SDI region.

In the 204 countries and territories in 2019, the highest ASRs of mortality and DALYs for pancreatic cancer for both sexes together were found in the United Arab Emirates and Uruguay, while the lowest rates (10 times lower) were reported in Ethiopia and Somalia. Also, there were apparent international variations in ASRs in both sexes together both for mortality and DALYs for pancreatic cancer that could be attributed to a high BMI in 2019: the highest ASR was found in the United Arab Emirates, while the lowest rate (100 times lower) was reported in Somalia. By GBD regions, the highest ASRs were observed in the region of Central Europe (in countries such as Czechia, Hungary, Montenegro, Serbia, Slovakia, Bulgaria, and North Macedonia) and the high-income North America region. Significant geographic variations in pancreatic cancer burden could be explained by differences in the age structure of the population in different countries, the prevalence of risk factors, as well as the improvements and availability of health care services [9,20,21,22,23]. Despite the improvements in many countries over the recent decades, it is known that life expectancy at birth in 2019 was the highest in countries in high-income GBD regions, and the lowest in Oceania, South Asia, and Africa [22]. Some variations in pancreatic cancer burden could be due to the differences in the implementation of prevention measures, improved therapeutic modalities, as well as in the practices of cancer certification and registration [21,22,23,24,25,26]. Also, certain other factors may contribute to the difficulties in international comparisons, including the frequency of autopsies, small population size, representation and heterogeneity in the histological subtypes, cohort effects, and some environmental risks. Among the risk factors associated with pancreatic cancer, the largest increase in risk exposure from 1990 to 2019 was described for a high body mass index [12,22]. This study indicated that 6.1% of the global pancreatic cancer burden in DALYs (5.1% in males, 7.5% in females) could be attributed to a high BMI in 2019, in contrast to 1990 (with a fraction of 4.8% in both sexes together, 3.8% in males vs. 6.2% in females). Also, in this study, all regions had a significant increase (*p* < 0.001) in pancreatic cancer burden (in mortality and DALYs) that could be attributed to a high BMI from 1990 to 2019. Moreover, this study showed consistently increasing trends in all age-specific rates for DALYs from pancreatic cancer that could be attributed to a high BMI from 1990 to 2019. Similarly, significantly increased trends of the percentage fraction in DALYs of pancreatic cancer that could be attributed to a high BMI were observed among all age groups in persons aged 20 years and over. Additionally, the increase in pancreatic cancer burden that could be attributed to a high BMI (for both sexes together and in all ages) across all GBD regions was significantly positively correlated with SDI from 1990 to 2019.

The prevalence of overweight and obesity has been increasing worldwide in recent decades, as well as the evidence of the rapid increase in prevalence and disease burden linked to elevated BMI [27,28]. The systematical evaluation of epidemiologic evidence about the causal relationship between a high BMI and pancreatic cancer among adults showed that the relative risk was 1.071 (95%CI = 0.999 to 1.154) in males and 1.092 (95%CI = 1.037 to 1.144) in females [28]. Although etiopathogenetic mechanisms that would explain the link between a high BMI and pancreatic cancer were not fully understood, some authors suggested that pancreatic fatty infiltration, chronic inflammation, altered cellular metabolism, hormone dysregulation, microbial dysbiosis, or immune cell infiltration may be involved in pancreatic carcinogenesis [29,30]. It is known that obesity favors the release of pro-inflammatory cytokines such as tumor necrosis factor α and Interleukin-6, which are linked to the development of inflammation, and the onset of cancer [31]. Also, one study found that fatty acid-binding protein 4 (that induces inflammation to promote cancer cell migration, invasion, and metastasis under obese conditions) was overexpressed in the serum of patients with obesity and was associated with poor overall survival in pancreatic cancer [32].

In this study, the global burden of pancreatic cancer that could be attributed to a high BMI in 2019 was higher in females than in males, in all ages. Also, the significantly unfavorable burden of pancreatic cancer that could be attributed to a high BMI was observed for both sexes in all ages globally, and across all SDI quintiles and all GBD regions. These findings may be partially explained by the differences in the prevalence rate of a high BMI in males and females. Some studies reported that females had a significantly higher rate of overweight and obesity than males [33]. The majority (>90%) of malignant tumors of the pancreas are pancreatic adenocarcinomas [34]. In the United States, the overall increases in the incidence and mortality of pancreatic cancer were primarily due to pancreatic adenocarcinoma [34,35]. The rising frequency of pancreatic adenocarcinoma in many high-income countries is linked to the changes in the prevalence of underlying risk factors, including the rising frequency of obesity, diabetes, and unhealthy dietary habits [36,37,38]. It is known that pancreatic adenocarcinoma is more common in males than females across various regions [1,39]. There is no difference in survival rates in pancreatic cancer by sex or geographical location [40]. Although the clinico-pathological features of cases with pancreatic adenocarcinoma under 50 years of age (early onset pancreatic cancer) are similar to the patients with this disease occurring after 50 years of age (late-onset pancreatic cancer), young patients were more frequently male, and had better overall survival [41]. Although continued efforts should be made to elucidate this, some studies reported that overweight or obesity during early adulthood was associated with a greater risk of pancreatic cancer and a younger age of disease onset [42,43].

In this study, the highest ASRs of pancreatic cancer burden were observed in the high SDI region in 2019. While a positive correlation between pancreatic cancer burden that could be attributed to a high BMI and SDI was observed in all GBD regions from 1990 to 2019, increases in pancreatic cancer burden attributable to a high BMI were faster in the low SDI region than in the high SDI region. Between 1990 and 2017, many countries/territories had a significant increase in the prevalence of a high BMI, whereby an increase was described even in low-income settings [44]. Differences in the growth and speed of growth of the prevalence of a high BMI worldwide as well as differences in the growth and speed of growth of the burden of pancreatic cancer attributable to a high BMI could be linked to the differences in socioeconomic development [45]. Namely, an increase in the level of national wealth can be the reason for an increased production and consequent increased food consumption and increased energy intake, which inevitably drive up the obesity frequency [46]. In addition, changes in the composition of age in a population, availability of improved diagnostics and advancements in the surveillance of cancers can partly explain the differences in the pancreatic cancer burden in countries with an increasing level of socioeconomic development [1]. Similar to this, recent studies suggest that the burden of pancreatic cancer in China is higher in regions with a higher level of urbanization, which could be linked to many factors related to overweight and obesity, including economic status, dietary choices, physical inactivity, and diabetes mellitus [47,48].

Apart from the abovementioned statements, the question remains regarding what the relative contribution to the global burden of pancreatic cancer is that could be attributed to a high BMI, which could be due to the differences between countries in applied prevention strategies, variations in inherited genetic factors, as well as possible exposure to some still unknown factors [27,28]. An open question remains whether the interaction effects of exposure to different factors (such as obesity, smoking, diet, etc.) on the burden of pancreatic cancer vary in different populations worldwide [9,10]. Therefore, there remains a need for further improvements in morphological diagnostics of pancreatic cancer, which would significantly contribute to a more precise assessment of the epidemiologic patterns between the many subtypes of cancer.

Since obesity was identified as an important risk factor for many cancers, numerous studies were conducted to evaluate how effective the therapies for cancer prevention (surgical or pharmacological) against obesity proved to be [49,50]. Weight reduction (which correlates with a low-fat and -sugar diet and a high dietary intake of fresh fruit and vegetables, and their associated nutrients like fiber, antioxidants, and polyphenols) together with a healthy lifestyle and regular exercise may contribute to the prevention of pancreatic cancer [50,51]. Some studies suggested that certain anti-inflammatory drugs (such as aspirin), anti-diabetic drugs (such as metformin), and anti-dyslipidemic drugs (such as statins) are considered as potential chemo-preventive agents for pancreatic cancer development, although the results from previous studies were inconsistent [52,53,54]. Although there is increasing evidence that suggests that bariatric surgery is associated with a reduced risk of pancreatic cancer, this protective effect may change with time, and further studies are needed to verify these results [55].

In summary, pancreatic cancer is becoming a more important contributor to the global cancer-related burden, with a continuous shift towards low-income countries. Moreover, marked international differences in the rates and trends in the burden of pancreatic cancer attributable to a high BMI indicate the need for an introduction of a more effective public health approach with regard to the prevention and improvements in diagnostic and treatment practices worldwide. Primary prevention has been shown to represent the key factor in reducing the burden of cancer. Certain risk factors for cancer, such as a high BMI, are preventable. To address this, a high BMI should be continuously evaluated and controlled, as well as all other modifiable risk factors including smoking and alcohol consumption. There remains a need for an introduction of a more effective public health approach regarding the prevention and diagnosis of pancreatic cancer worldwide, including a further implementation of a healthy lifestyle, particularly physical activity and a proper diet choice as they are important factors that can lower a person’s BMI.

This study analyzed global patterns in the trends of the pancreatic cancer burden from 1990 to 2019, as well as the contribution of a high BMI to the burden of pancreatic cancer worldwide and their association. Moreover, this study presented estimates of the pancreatic cancer burden for 204 countries and territories using high-quality data from the GBD 2019 study. Additionally, the joinpoint analysis of the trends allowed for an accurate interpretation of the changes over time and to determine if these changes were statistically significant. However, some limitations should be considered. First of all, the question of reliability, accuracy, and coverage of pancreatic cancer mortality data can always be raised. Furthermore, another limitation of this study is the inability of conducting a more detailed analysis due to the lack of data on the anatomical locations and histological subtypes of pancreatic cancer within subjects. Furthermore, the issue of underreporting and the availability of data on the frequency of pancreatic cancer and a high BMI, especially in low-income countries, may result in a bias in the assessment of trends in the burden of pancreatic cancer in the world. In addition, by including the latest available data in this analysis, our study could not provide estimates of the pancreatic cancer burden for the period of the COVID-19 pandemic. Moreover, this study did not analyze some potential covariates, such as diabetes mellitus, alcohol use, etc. Finally, the fallacy inherent to the ecological design of this study is an important limitation of this study. Therefore, the results of this study on the association between pancreatic cancer burden and a high BMI must be elucidated in analytical longitudinal studies.

## 5. Conclusions

Pancreatic cancer burden became a major challenge to public health. Increasing trends in pancreatic cancer burden that could be attributed to a high body mass index were observed in all regions from 1990 to 2019, particularly in low-income countries. Further analytical epidemiological research is necessary to explain the relationship between pancreatic cancer and a high body mass index.

## Figures and Tables

**Table 1:** Deaths of pancreatic cancer attributable to a high body mass index, in all ages, by locations, 1990–2019; a joinpoint regression analysis.

	Deaths in 1990	ASR in 1990	Deaths in 2019	ASR in 2019	AAPC (ASR) (95%CI)	*p* Value
Global						
Male	3841	0.22	13,598	0.36	1.7 * (1.7 to 1.8)	<0.001
Female	5851	0.28	18,323	0.42	1.3 * (1.3 to 1.3)	<0.001
Both sexes	9693	0.26	31,921	0.40	1.4 * (1.4 to 1.5)	<0.001
― SDI regions						
High SDI	4945	0.47	12,901	0.67	1.3 * (1.2 to 1.3)	<0.001
High–middle SDI	3635	0.35	10,576	0.52	1.3 * (1.2 to 1.4)	<0.001
Middle SDI	834	0.09	5974	0.24	3.7 * (3.6 to 3.8)	<0.001
Low–middle SDI	207	0.04	2005	0.15	5.0 * (4.9 to 5.1)	<0.001
Low SDI	69	0.03	449	0.09	4.1 * (3.9 to 4.2)	<0.001
― GBD regions						
Andean Latin America	21	0.10	239	0.43	5.3 * (4.6 to 6.0)	<0.001
Australasia	110	0.47	347	0.69	1.4 * (1.3 to 1.4)	<0.001
Caribbean	22	0.09	201	0.39	5.1 * (4.3 to 5.9)	<0.001
Central Asia	68	0.15	334	0.48	5.0 * (4.5 to 5.4)	<0.001
Central Europe	919	0.62	2083	0.97	1.6 * (1.5 to 1.7)	<0.001
Central Latin America	219	0.27	995	0.43	1.3 * (1.2 to 1.5)	<0.001
Central Sub-Saharan Africa	13	0.06	51	0.10	1.4 * (0.8 to 2.0)	<0.001
East Asia	484	0.06	4402	0.21	4.9 * (4.7 to 5.2)	<0.001
Eastern Europe	1365	0.48	2593	0.75	1.3 * (1.1 to 1.6)	<0.001
Eastern Sub-Saharan Africa	26	0.04	181	0.11	4.4 * (4.1 to 4.6)	<0.001
High-income Asia-Pacific	452	0.23	1315	0.28	0.6 * (0.5 to 0.7)	<0.001
High-income North America	2126	0.60	5814	0.91	1.5 * (1.4 to 1.6)	<0.001
North Africa and Middle East	277	0.17	2006	0.48	3.8 * (3.6 to 4.0)	<0.001
Oceania	2	0.08	9	0.14	1.6 * (1.3 to 1.9)	<0.001
South Asia	103	0.02	1393	0.10	6.0 * (5.8 to 6.2)	<0.001
Southeast Asia	98	0.04	990	0.16	5.2 * (5.1 to 5.2)	<0.001
Southern Latin America	204	0.45	671	0.80	1.8 * (1.5 to 2.1)	<0.001
Southern Sub-Saharan Africa	79	0.30	300	0.57	2.2 * (1.9 to 2.4)	<0.001
Tropical Latin America	233	0.27	1197	0.50	2.3 * (2.3 to 2.4)	<0.001
Western Europe	2814	0.48	6336	0.67	1.2 * (1.1 to 1.3)	<0.001
Western Sub-Saharan Africa	55	0.06	465	0.26	5.0 * (4.9 to 5.0)	<0.001

ASR = age-standardized rates (per 100,000); for the full period (1990–2019) presented AAPC = average annual percentage change; 95% CI = confidence interval; SDI = socio-demographic index. * Statistically significant trend (*p* < 0.05). Source: Global Burden of Disease Study [14].

**Table 2:** DALYs of pancreatic cancer attributable to high body mass index, in all ages, by locations, 1990–2019; a joinpoint regression analysis.

	DALYs (Number) in 1990	ASR in 1990	DALYs (Number) in 2019	ASR in 2019	AAPC (ASR) (95%CI)	*p* Value
Global						
Male	98,571	5.12	329,455	8.27	1.7 * (1.6 to 1.7)	<0.001
Female	125,683	5.90	379,994	8.69	1.3 * (1.3 to 1.3)	<0.001
Both sexes	224,255	5.60	709,449	8.54	1.4 * (1.4 to 1.5)	<0.001
― SDI regions						
High SDI	106,212	10.37	256,852	14.62	1.2 * (1.2 to 1.3)	<0.001
High–middle SDI	87,986	8.01	238,575	11.64	1.2 * (1.1 to 1.3)	<0.001
Middle SDI	22,529	2.04	150,391	5.74	3.7 * (3.6 to 3.7)	<0.001
Low–middle SDI	5555	0.87	51,063	3.57	5.1 * (5.0 to 5.1)	<0.001
Low SDI	1892	0.74	12,202	2.18	4.0 * (3.9 to 4.2)	<0.001
― GBD regions						
Andean Latin America	552	2.57	5611	9.88	5.0 * (4.3 to 5.6)	<0.001
Australasia	2420	10.39	6860	14.67	1.3 * (1.2 to 1.4)	<0.001
Caribbean	540	2.05	4625	8.90	5.1 * (4.3 to 5.9)	<0.001
Central Asia	1774	3.65	8790	11.11	4.8 * (4.3 to 5.3)	<0.001
Central Europe	22,298	14.87	45,286	22.20	1.4 * (1.3 to 1.5)	<0.001
Central Latin America	5659	6.44	23,811	9.88	1.2 * (1.1 to 1.4)	<0.001
Central Sub-Saharan Africa	360	1.42	1455	2.44	1.4 * (0.8 to 2.0)	<0.001
East Asia	13,477	1.41	110,235	5.11	4.8 * (5.6 to 5.0)	<0.001
Eastern Europe	34,253	12.00	60,662	18.00	1.1 * (0.8 to 1.4)	<0.001
Eastern Sub-Saharan Africa	733	0.89	5041	2.80	4.3 * (4.1 to 4.6)	<0.001
High-income Asia-Pacific	10,336	5.02	23,154	5.70	0.4 * (0.3 to 0.4)	<0.001
High-income North America	46,249	13.74	120,686	20.03	1.3 * (1.2 to 1.4)	<0.001
North Africa and Middle East	7527	4.06	52,345	11.26	3.6 * (3.4 to 3.8)	<0.001
Oceania	70	2.07	267	3.35	1.4 * (1.1 to 1.8)	<0.001
South Asia	2824	0.46	35,432	2.41	6.0 * (5.9 to 6.2)	<0.001
Southeast Asia	2855	0.99	26,486	4.02	4.9 * (4.8 to 5.0)	<0.001
Southern Latin America	106,212	9.98	14,078	17.12	1.7 * (1.4 to 2.0)	<0.001
Southern Sub-Saharan Africa	87,986	7.10	7318	12.58	2.0 * (1.7 to 2.2)	<0.001
Tropical Latin America	22,529	6.21	27,596	11.18	2.2 * (2.1 to 2.3)	<0.001
Western Europe	5555	10.42	117,439	14.10	1.1 * (1.0 to 1.2)	<0.001
Western Sub-Saharan Africa	1892	1.55	12,271	6.12	4.8 * (4.7 to 4.9)	<0.001

DALYs = Disability-Adjusted Life Years; ASR = age-standardized rates (per 100,000); for the full period (1990–2019) presented AAPC = average annual percentage change; 95% CI = confidence interval; SDI = socio-demographic index. * Statistically significant trend (*p* < 0.05). Source: Global Burden of Disease Study [14].

## Data Availability

Data are contained within this article.
